# Anything is better than nothing’: exploring attitudes towards novel therapies in leukodystrophy clinical trials

**DOI:** 10.1186/s13023-024-03320-9

**Published:** 2024-09-05

**Authors:** Ella Wilson, Richard Leventer, Chloe Cunningham, Michelle G. de Silva, Jan Hodgson, Eloise Uebergang

**Affiliations:** 1 Murdoch Children’s Research Institute, Melbourne, VIC Australia; 2 Australian Genomics, Melbourne, VIC Australia; 3 Royal Children’s Hospital, Melbourne, VIC Australia; 4 Victorian Clinical Genetics Services, Melbourne, VIC Australia; 5 The University of Melbourne, Melbourne, VIC Australia

**Keywords:** Leukodystrophy, Clinical-trials, Rare disease, Attitudes, Novel therapies, Gene therapies, White matter disorders, Pediatric neurology, Adult neurology

## Abstract

**Background/Aim:**

Leukodystrophies comprise a group of genetic white matter disorders that lead to progressive motor and cognitive impairment. Recent development of novel therapies has led to an increase in clinical trials for leukodystrophies. To enable recruitment of individuals with a leukodystrophy into clinical trials, clinical trial acceptability should be ascertained. We sought therefore, to identify the motivations for and barriers to clinical trial participation in addition to clinical trial features that may be of concern to individuals with a leukodystrophy and/or their carers.

**Methods:**

Adults with a leukodystrophy and parents/carers of individuals with a leukodystrophy were recruited through the Australian Leukodystrophy Registry and through online advertisements. Qualitative semi-structured interviews were used to explore participants views on what clinical trials involve, the perceived risks and benefits of clinical trials, their desire to participate in clinical trials and their personal experience with leukodystrophy. Thematic analysis of data was performed with co-coding of interview transcripts.

**Results:**

5 interviews were held with parents of children with leukodystrophy, 4 with parents of adults with leukodystrophy and 3 with adults diagnosed with leukodystrophy. Motivations for clinical trial enrolment include access to potentially lifesaving novel treatments and improved prognostic outcomes. Participants were concerned about adverse clinical trial outcomes, including side effects and exacerbation of illness. Despite this, majority of participants were willing to try anything in clinical trials, demonstrating a high tolerance for first in human trials and trials utilising invasive treatment options.

**Conclusions:**

Interviewees communicated a strong desire to participate in interventional clinical trials involving novel therapies. To support enrolment into future leukodystrophy clinical trials we suggest the provision of transparent information regarding clinical trial treatments, consideration of alternative trial control measures, and inclusion of treating clinicians in the trial recruitment process. Clinicians play an integral role in initiating transparent conversations regarding trial risks and adverse outcomes.

## Background

Leukodystrophies comprise a group of over 30 rare and progressive genetic disorders that affect the Central Nervous System (CNS) white matter [1]. Collectively, leukodystrophies affect approximately 1/7000 individuals [2].

With advances in next-generation sequencing technology, many leukodystrophies are now classified according to their underlying genetic pathology [3–5]. The discovery of candidate Leukodystrophy genes has allowed for development of targeted novel therapies [4, 6] which are predominantly gene and RNA based [7, 8].To assess the efficacy of prospective therapies, treatment focused Leukodystrophy clinical trials continue to emerge [4, 9]. In order to support enrolment into clinical trials, an understanding of the attitudes held by the relevant disease community towards interventional clinical trials is crucial. Whilst clinical trial acceptability in individuals with Leukodystrophy and their carers remain unknown, attitudes towards clinical trials have been examined in several other rare disease populations.

### Defining patient attitudes

The term ‘patient attitudes’ refers broadly to a patients’ knowledge of and perception towards clinical trials [10, 11]. To understand and measure patients’ attitudes in this research context, previous studies have examined motivations for and barriers to trial participation and patients’ willingness to participate in clinical trials [10, 12–14].

### Motivations for clinical trial participation

It is well understood that patients’ motivation to participate in interventional clinical trials is driven by a perceived benefit to their health and altruistically the health of others [12, 13]. Further motivations driving participation include patients’ hopes to improve clinical understanding of their condition [14] and to increase awareness of their condition [12].

It is notable that altruistic motivations for clinical trial participation appear to be more prominent in rare and severe pediatric disease populations [15, 16]. In 2012, a systematic review [15] explored parental decision-making for enrolment of children into rare disease clinical trials. They found that a key motivating factor for clinical trial participation was to help improve health outcomes of children diagnosed in the future [15]. Comparatively, in more common, less severe disease populations (e.g., Type 2 diabetes and lupus) greater emphasis is placed on the benefit that clinical trial participation brings to the individual [17]. Although motivations for enrolment remain unknown in the leukodystrophy population, such findings suggest that altruistic outcomes may be a key driver for participation in leukodystrophy clinical trials.

### Barriers to clinical trial participation

The barriers to clinical trial participation have been well characterised in cancer populations, and less so in rare disease groups [14]. Several studies have however reached consensus for the main deterrents preventing rare disease patients’ from enrolling in clinical trials. These include risk of side effects, burden of travel and the potential requirement to stop current medication [13, 17].

### Clinical trial design features and willingness to participate

Clinical trial design has a crucial influence over patients’ willingness to enrol and participate in clinical trials [18, 19]. The phase assigned to a clinical trial (Phase I, Phase II or Phase III) appears to be an important consideration for prospective participants. Phase I clinical trials, some of which may be ‘first in human’ trials [20] are perceived by patients’ to incur greater risk, with preference being given to Phase II clinical trials [21]. These results appear consistent in rare disease populations, as shown in patients’ with Fredrich Ataxia [13] and Huntington Disease [22].

The nature of the clinical trial intervention has also been reported to influence patients’ willingness to participate. Invasive interventions appear to act as deterrents, with preference given to non-invasive orally administered therapies [19]. The inclusion of placebo groups helps to evaluate the efficacy of active treatment interventions [21]. From the patients’ perspective however, the chance of random assignment to a placebo group can act as a deterrent for clinical trial enrolment [13, 17].

Whilst clinical trial features have been shown to influence participation in other rare disease trials, the acceptability for clinical trial design features in leukodystrophy patients’ remains unknown. This study sought to address this gap in research by exploring the attitudes of individuals with leukodystrophy or their carers towards clinical trials.

## Methods

### Study design

Using qualitative methodology, the perspectives of individuals with leukodystrophy and their carers were captured through exploratory semi-structured interviews. Interviews explored participants understanding of the risks and benefits of clinical trials, their concerns and expectations, motivations for enrolment into clinical trials and the impact of clinical trial design features on willingness to participate.

### Recruitment and participant characteristics

Our study population included adults diagnosed with a leukodystrophy and the carers/parents of children and adults diagnosed with a leukodystrophy. Leukodystrophy diagnoses were confirmed by clinical members of the research group (geneticist and neurologist) based on prior imaging and clinical features.

**Fig. 1:**
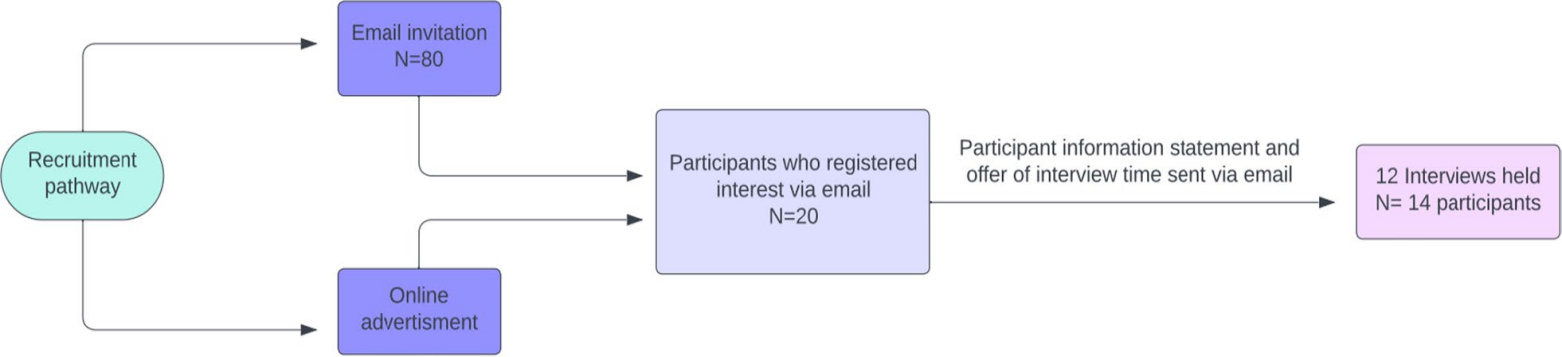
Recruitment strategy

Participants were purposefully sampled by way of email invitation and online advertisement through the Australian Leukodystrophy registry (https://www.leukonet.org.au/patient-registry/) and social media of Leukodystrophy Australia and Mission Massimo foundation. Prospective participants who registered interest via email were provided a participant information statement and offer of time for formal interview (Fig. 1).

### Data collection

Recorded semi-structured interviews were held between April–July 2022. All participants were given the opportunity to discuss the study and ask the primary researcher questions prior to agreeing to take part. Interviews (N = 12) were conducted in person (N = 1), via telephone (N = 1) and via Zoom (N = 10) and recorded via Dictaphone or videorecording. Demographic information and verbal consent for participation was recorded at the beginning of each interview. Recordings were transcribed verbatim with all identifying information redacted prior to analysis.

### Analysis

Interview transcripts were read by the primary researcher with initial notes and reflections made. A data-driven approach was then used for coding of transcripts through QSR Internationals NVivo software. Broad top-level codes were initially generated with addition of child nodes as analysis progressed. Four interviews were independently co-coded by three members of the research team, with comparison made between initial themes generated. Themes were reviewed and refined through discussion between the wider research group, until consensus was reached.

## Results

Fourteen participants took part in twelve interviews, five interviews held with parents of children with leukodystrophy, four with parents of adults with leukodystrophy, and three with adults with leukodystrophy. Participants were characterised as being the parent/carer of or individual diagnosed with leukodystrophy, sex, age at time of interview, age of Leukodystrophy diagnosis, type of leukodystrophy diagnosis and stage of disease. See Table 1 for demographic characteristics.

**Table 1:** Participant demographic characteristics

Participant type	Sex	Age at interview (years)	Age at diagnosis (a)	Type of Leukodystrophy (b)	Interview length (minutes)	Stage of disease
Parent Child with Leukodystrophy	Female Male	50–55 15–20	– 4 years	– Unclassified	51	Late stage
Parent Child with Leukodystrophy	Female Female	40–45 10–15	– 9 years	– Ultra-rare*	53	Middle stage
Adult with Leukodystrophy	Male	40–45	40 years	Alexander Disease,adult-onset	54	Early stage
Adult with Leukodystrophy	Male	40–45	38 years	Alexander Disease,adult-onset	54	Early stage
Parent Adult with Leukodystrophy	Female Male	75–80 45–50	– 39 years	– Adrenoleukodystrophy, adult onset	36	Middle stage
Adult with Leukodystrophy	Male	35–40	30 years	Adrenoleukodystrophy, adult onset	50	Middle stage
Parent Adult with Leukodystrophy	Female Female	70–75 45–50	– 26 years	– Ultra rare* adult onset	– 32	Middle stage
Parent Adult with Leukodystrophy	Female Female	60–65 25–30	– 19 years	–	44	Middle stage
Parent Child with Leukodystrophy	Female Female	35–40 5–10	– Infancy	– Metochromatic leukodystrophy	37	Middle stage (stable)
Parent Guardian Child with Leukodystrophy	Male Female Male	35–40, 20–25	– – 19mo	*–* *–* Ultra-rare*	62	Middle stage (stable)
Parent Child with Leukodystrophy	Male Female	50–55 5–10	9 years	– Ultra-rare*	67	Early stage
Parent Adult with Leukodystrophy	Female Male	50–55 20–25	– 14 years	Alexander disease, juvenile onset	25	Late stage

Age at diagnosis refers to the individual diagnosed with a Leukodystrophy

To protect anonymity of participants with ultrarare Leukodsytrophy the specific diagnosis has been omitted. Unclassified Leukodystrophy refers to a diagnosis of Leukodystrophy for which a causal genetic basis has not been identified

^*^Ultrarare Leukodystrophy is defined as prevalence of less than 1/50,000 affected individuals [23]

Disease stage as self-reported or described by treating clinician

**Fig. 2:**
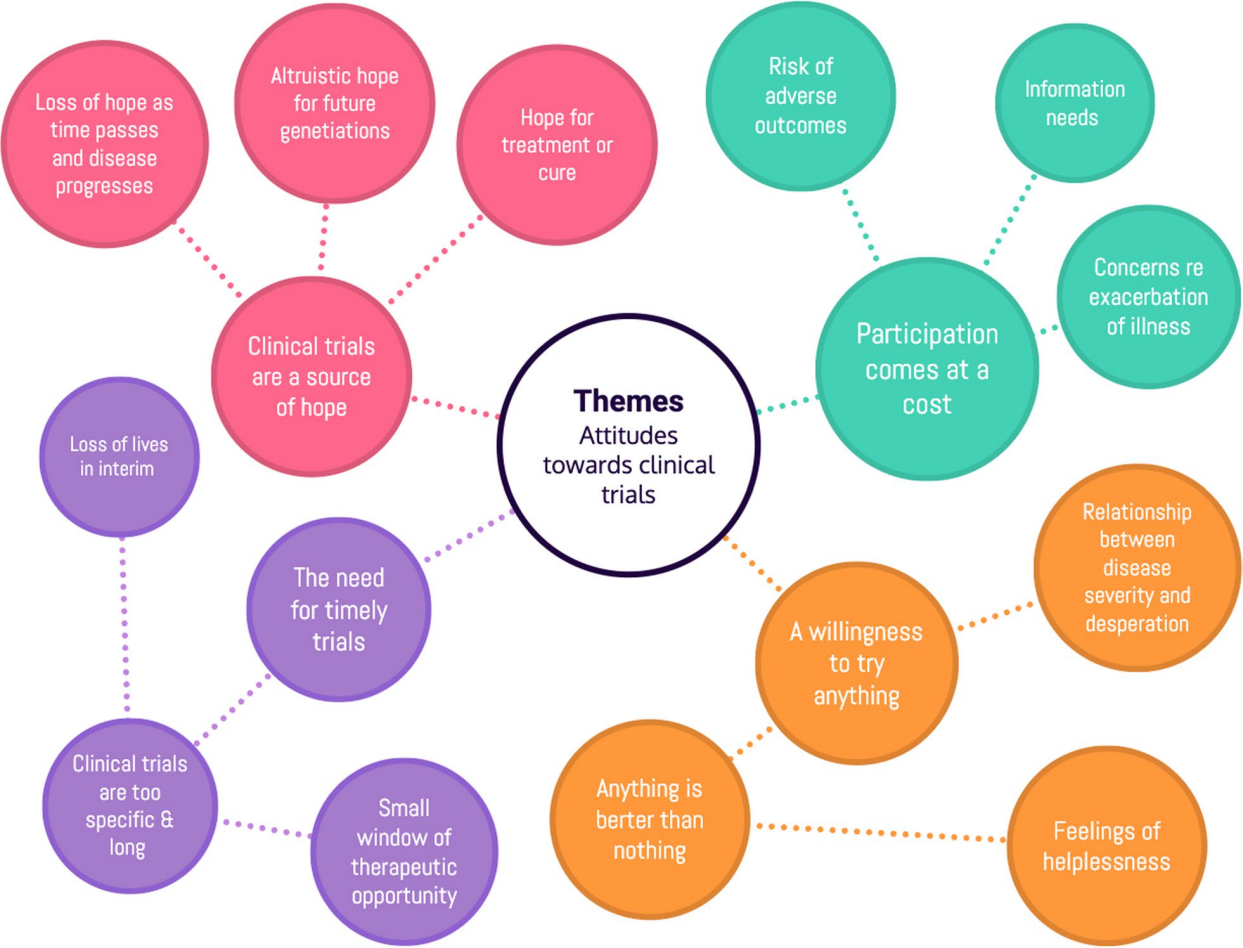
Mind map of themes identified through participant interviews

The themes illustrated below (Fig. 2) capture the shared perceptions held by individuals with leukodystrophy and their carers, towards clinical trials.

### Patient knowledge of Leukodystrophies and clinical trials

Participants well defined leukodystrophies and referenced the molecular basis underlying theirs or their childs diagnosis. Interviewees used technical terms when describing disease mechanisms, including *“destruction of the myelin sheath”, “inability to breakdown long chain fatty acids*” and the *“absence of enzyme arylsulfatase A”*. They found difficulty obtaining prognostic information and frequently sought out journal articles targeted at leukodystrophy academics and clinicians. Participants were able to describe the broad nature of interventional clinical trials, and all had researched past and present trials specific to their or their child’s diagnosis. Only one interviewee had successfully gained access to a clinical trial despite several having tried.

### Hope as a motivator for clinical trial enrolment

Collectively interviewees described the prospect of trying novel treatments to be a source of hope in the face of an otherwise ‘hopeless’ diagnosis. They hoped treatments in a trial would improve quality of life, slow or halt disease progression and altruistically reduce suffering in future individuals diagnosed with leukodystrophy. Some participants described a hopeful yet realistic approach to clinical trial outcomes with others describing grand hopes for curative treatment.

*‘Well, my hope is not to reverse it, but to stop it from getting any worse. You know, plateauing, whether it be week by week, year by year, month by month. That you won’t get any worse. To reverse it is a big call. But to subside it.’ –* Adult with leukodystrophy.

‘*There are people that have been told they can't walk again, and suddenly, somehow, they walk again. And the brain is an amazing thing. Sometimes it can do things, you didn't think it could do and it's just about finding the right trigger for it to do it. So, the motivation, to some degree is that silver bullet.’* – Parent of child with leukodystrophy.

Length of time since diagnosis appeared to influence participants levels of hope, with those diagnosed longer ago seemingly having less hope tied to clinical trials.

‘*I don't know what every parent says but there is still hope for a cure…maybe it’s not realistic, but it's not beyond the realms of complete possibility.—*Parent of child with leukodystrophy, diagnosed 12 months ago.

*‘I would use the term hope very loosely. To me knowing what’s out there (clinical trials) seems better than blind ignorance to the situation.’-* Adult with leukodystrophy, diagnosed 9 years ago.

*‘It is hard to think of anything that could improve him. You don’t really go there as a parent.’ –* Parent of adult with leukodystrophy, diagnosed 15 years ago.

### A willingness to try anything

Twelve of fourteen participants spoke of a willingness to try ‘anything’ with proposed therapeutic benefit in clinical trial, in preference to the current alternative of no treatment. For most participants, the willingness to try anything stemmed from feelings of helplessness. Parents of children with leukodystrophy felt a sense of control may be gained by taking action to enrol their child in a clinical trial and that this would allow them to reflect on their child’s diagnosis knowing every attempt was made to improve their quality of life.

Most participants were accepting of control measures in clinical trials, and several demonstrated a high tolerance for experimental and invasive treatment options including spinal and intracerebral injections.

*‘I have been poked and prodded a hell of a lot over the journey so to me it makes little difference whether it (treatment) be a pill or something more extreme like a spinal injection.’ –* Adult with leukodystrophy.

Several participants raised concerns regarding possible adverse treatment outcomes from first in human trials but were clear that this would not deter them from enrolment.

‘*They are trialling it in the human body for the first time, and the body is very complicated, complex… Anything could happen, but I would quite comfortably give it ago because there is no other option.’ –* Adult with leukodystrophy.

Most participants appeared willing to participate in placebo-controlled trials and recognised a placebos role in establishing the effectiveness of a gene therapy or medication. Notably,

we found that the parents of the only two children with clinically stable (non-progressive) leukodystrophy were less tolerant for the use of placebos and invasive treatment options.

‘*If it were a possible trial with a placebo and it involved injections or hurting her in any way, then no, I wouldn’t consider participation’. –* Parent of child with stable leukodystrophy.

*‘Unless it was to rebuild the white matter in his brain, I probably wouldn’t put him through any of it since nothing is actively happening. If he was still progressing, then we would try anything.’*

*–* Parent of child with stable leukodystrophy.

### The need for timely trials

Due to the progressive and unrelenting course of leukodystrophies, participants raised concerns regarding current Leukodystrophy clinical trial timelines.

*‘It would be nice that if you took a chance on something and it did work, it slowed things down sooner rather than later. As opposed to waiting another three years for the trial to finish and then transitioning to whatever it is that has been trialled. I guess, cause time isn’t on our sideyou know.’ –* Adult with leukodystrophy.

Participants frequently commented on the length and estimated completion dates of currently active leukodystrophy clinical trials, with concerns regarding lives lost in the interim. Participants also highlight the need for novel treatments to be made available prior to the onset of theirs or their child’s irreversible white matter damage.

*‘There are timings for things as well. A treatment given too late is a wasted treatment.’ –* Parent of Adult with leukodystrophy.

One participant had recently been unsuccessful in gaining access to a novel antisense oligonucleotide therapy clinical trial. They emphasised the importance of open communication and transparency from clinical trial recruiters and the role this plays in managing patient expectations of clinical trial outcomes.

‘*One of the problems is a lack of communication. I get they don’t want to jeopardize the trial by sharing information, but still these patients’, parents, people like me who are affected, we get discouraged. We know that we have a disease, it is like a timebomb ticking away that it is going to explode soon.–* Adult with leukodystrophy.

Participants made the following suggestions for clinical trials to better meet their time sensitive needs: Increase participation numbers, broaden trial selection criteria and exclude placebos where possible.

### Participation comes at a cost

Overall, all fourteen participants’ attitudes towards clinical trials were overwhelmingly positive, although concerns were communicated regarding potential for novel therapies to exacerbate or accelerate illness.

*‘A risk I envisage is the possibility of your situation getting worse, or the progress of the disease getting sped up.’ –* Adult with leukodystrophy.

Further, concerns regarding adverse clinical trial outcomes, side effects and infliction of pain were referenced.

*‘I suppose the major risk would be an adverse reaction which could either one, make the condition worse, or two result in end of life. –* Parent of child with leukodystrophy.

One parent whose child had become critically ill after receiving an experimental treatment in trial, reflected on feeling blind to the seriousness of said trial risks. They highlight the need for trial staff to appropriately emphasise risk and ensure this not be overshadowed by what participants perceive to be positive outcomes.

*‘Coming in they sat us down and went, well this thing could go wrong, and this thing could go wrong, and it felt like the kind of conversation you would have if you were going in for minor surgery…and then when we were in there it’s not a ‘there’s a small chance this could go wrong’ it’s a ‘there’s 30% chance this could go wrong’… I guess we went into it a little bit naively.’*

Interviewees felt they would be less concerned to enrol in a clinical trial if provided adequate and transparent information during the recruitment phase. Their key information needs ﻿are described below (Table 2).

**Table 2:** Example quotes: informational needs of prospective trial participants

Informational needs	Representative quotes
Tolerability and origin of treatment	*‘I like to know what it does, and why it does what it intended to do, and how it makes changes to improve whatever it is you're trying to resolve.’—*Adult with leukodystrophy *‘I would like to know where it’s (the drug or treatment) coming from* *What is it? At the moment the supplement she is on is the bile from an Ox.’* *–* Parent of adult with leukodystrophy
Time commitment and requirement to travel	*‘I would want to know about the frequency of a dose and duration (of a trial), and whether it can be administered rurally or if we would have to travel to the city.’—*Parent of child with leukodystrophy *‘I’d like to know how often we’d need to go into the hospital. I am on a carer’s pension and so I have to budget for parking whenever (x) has medical appointments.’—*Parent of adult with leukodystrophy
Anticipated adverse outcomes	*‘Uh, I guess I would like any medical procedure, um, to know the possibilities or complications that may happen due to that being administered.’ –*Parent of child with ultra-rare leukodystrophy *‘You know, for myself I want to make an informed decision on things… If you could lose a leg out of this or risk amputation that’s not something to skim over.’—*Adult with leukodystrophy

They also felt that the support of their trusted treating clinicians would further minimise concerns associated with clinical trial participation.

*‘I would really respect that geneticist’s input—you know whether, she said it (the clinical trial) was going to be okay. I would really take that on board and feel reassured’.—*Parent of adult with leukodystrophy.

*‘There is no doubt I’d involve his treating team. If they are involved, they can probably dumb down some stuff for me. You know there might be some things I look at and think ‘that’s fine’ but they say hang on, this isn’t very good.’ –* Parent of child with leukodystrophy.

### Attitudes in self reporting adults versus caregivers

Of three self-reporting adult participants two were diagnosed with adult-onset Alexander disease, one with adult onset adrenoleukodystrophy. Attitudes towards trial design features and concerns about trial length were consistent between self-reporting adults and caregivers, although self-reporting adults expressed more concerns with trials disrupting daily life and work schedules.

## Discussion

This study examined clinical trial acceptability in individuals with leukodystrophy with the aim to support enrolment into future leukodystrophy clinical trials and optimise trial participant experience.

### Willingness to participate in leukodystrophy clinical trials

Participants communicated a strong desire to participate in clinical trials, with hope for better prognostic outcomes being a key motivator. Altruistically, parents of children referenced the difficulty in watching their child suffer and hoped that participation in clinical trials may also prevent such suffering for future children and their parents. Such findings have been observed in other paediatric rare disease cohorts [12, 15, 24], suggesting that parents in rare disease communities have a strong desire to support one another and in part do so by participating in clinical trials.

Adults with leukodystrophy and parents of children with leukodystrophy demonstrated a high tolerance for experimental measures in clinical trials, a reflection of their desperation to access treatment. Clinical trial design features appeared to have minimal influence on willingness to participate, with most participants demonstrating a high tolerance for trials utilising invasive treatment interventions such as lumbar punctures and intraparenchymal brain injections. Further, our participants appeared highly willing to partake in first in human trials [25], despite voicing concerns regarding possible adverse outcomes.

When describing their perception of clinical trials, interviewees had a tendency focus on the benefits of clinical trials, including the opportunity to access potentially lifesaving treatment. Participants made less frequent references to clinical trial risks, and usually compared these to their or their child’s already poor prognosis. The propensity to emphasise positive clinical trial outcomes and minimise focus on possible adverse outcomes has been observed in other patients with neurodegenerative or rare diseases [16, 26, 27]. This phenomenon known as ‘therapeutic optimism’ is an important consideration for leukodystrophy clinical trial educators and clinicians, who play a role in management of patient expectations and ensuring participants appropriate perception of risk.

Clinicians also play an important role in advising and supporting patients and their carers who are considering entering a clinical trial. To avoid bias, it is ideal that the physician providing clinical care is not the principal trial investigator, although this is not always possible for rare diseases where disease expertise is limited. Provision of unbiased, objective information regarding potential side effects and risks of participating in a clinical trial is critical. Although anything *may* be better than nothing for individuals with progressive neurological disorders, patients and carers must be reminded that involvement in the trial itself, particularly in the early safety and tolerability phases, may accelerate the disease or result in serious side effects that would not have occurred without trial involvement.

### Managing patient expectations in leukodystrophy clinical trials

Several participants raised concerns regarding timelines of clinical trials, highlighting the time pressures associated with progressive neurodegenerative disease. Interviewees referenced timeframes for existing and current leukodystrophy clinical trials [28] (Clinical Trial identifier: NCT01560182; Clinical Trial identifier: NCT04849741) and were forthright in stating that patients’ would die before prospective treatments in trial were brought to market. Similar concerns have been echoed by adults and parents of other children with rapidly progressive disorders [12, 29], recognising the need to access treatment before the window of therapeutic opportunity is lost. To address timeline concerns for rare and progressive diseases, pharmaceutical regulation agencies have begun to expedite new therapies for rare diseases to market by introduction of accelerated pathways for orphan drug approval [30].

For patients who do not fulfil strict inclusion criteria for participation in leukodystrophy trials, compassionate access should be considered to ensure they have timely and equitable access to investigational treatments that may be of therapeutic value [31]. Compassionate use has been strongly advocated by leukodystrophy clinicians, as per formal recommendations for trial design recently published by the Vanishing White Matter (VWM) Disease Consortium [32]. In addition to fast-track approval and compassionate drug access, clinical trial providers can further manage patient expectations by providing transparent information regarding trial timelines and avenues to access novel treatments that have proved efficacious in trials.

### Addressing attitudes towards trial design

Whilst most participants were understanding of the need for placebo-controls in trials all agreed it would be favourable for trial participants to be guaranteed provision of the active treatment. Such views have been repeatedly echoed by members of rare disease cohorts [17, 33]. This raises challenges for clinical trial providers, who must achieve rigor in trial design to ensure validity of clinical trial outcomes [29, 34]. The use of control measures in rare disease trials is particularly important, given the typically small sample size [35] and spectrum of phenotypic variability observed between individuals who may be at different stages of disease progression [34]. However, some adjustments to clinical trial design in rare disease cohorts have enabled providers to meet patient needs, without compromising clinical trial rigour [34]. For conditions with well-established natural history studies, the replacement of a placebo arm with a natural history arm, is an effective and ethically preferable solution [31, 34]. Crossover arms in which study participants are assigned two groups with dual phases (one placebo, one active) have also been used in rare disease cohorts in preference to placebo arms, as have integration of open label extension phases [33].

### Facilitating recruitment into leukodystrophy clinical trials

Participants placed great value on honesty and transparency from clinical trial recruiters. Unsurprisingly, the establishment of participant trust through honest and transparent communication from clinical trial recruiters has proven integral for participant enrolment and retention in clinical trials of all nature [36, 37].

Those participants who described trusting relationships with treating clinicians, felt that their support and involvement would positively influence their decision to enrol in a clinical trial. The recommendation and support for clinical trials from treating clinicians has proven to be a key motivating factor for clinical trial enrolment in other disease groups [13, 38]. The endorsement of clinical trials by treating clinicians requires careful consideration however, to ensure decision making regarding trial enrolment of patients remains autonomous [39].

**Fig. 3:**
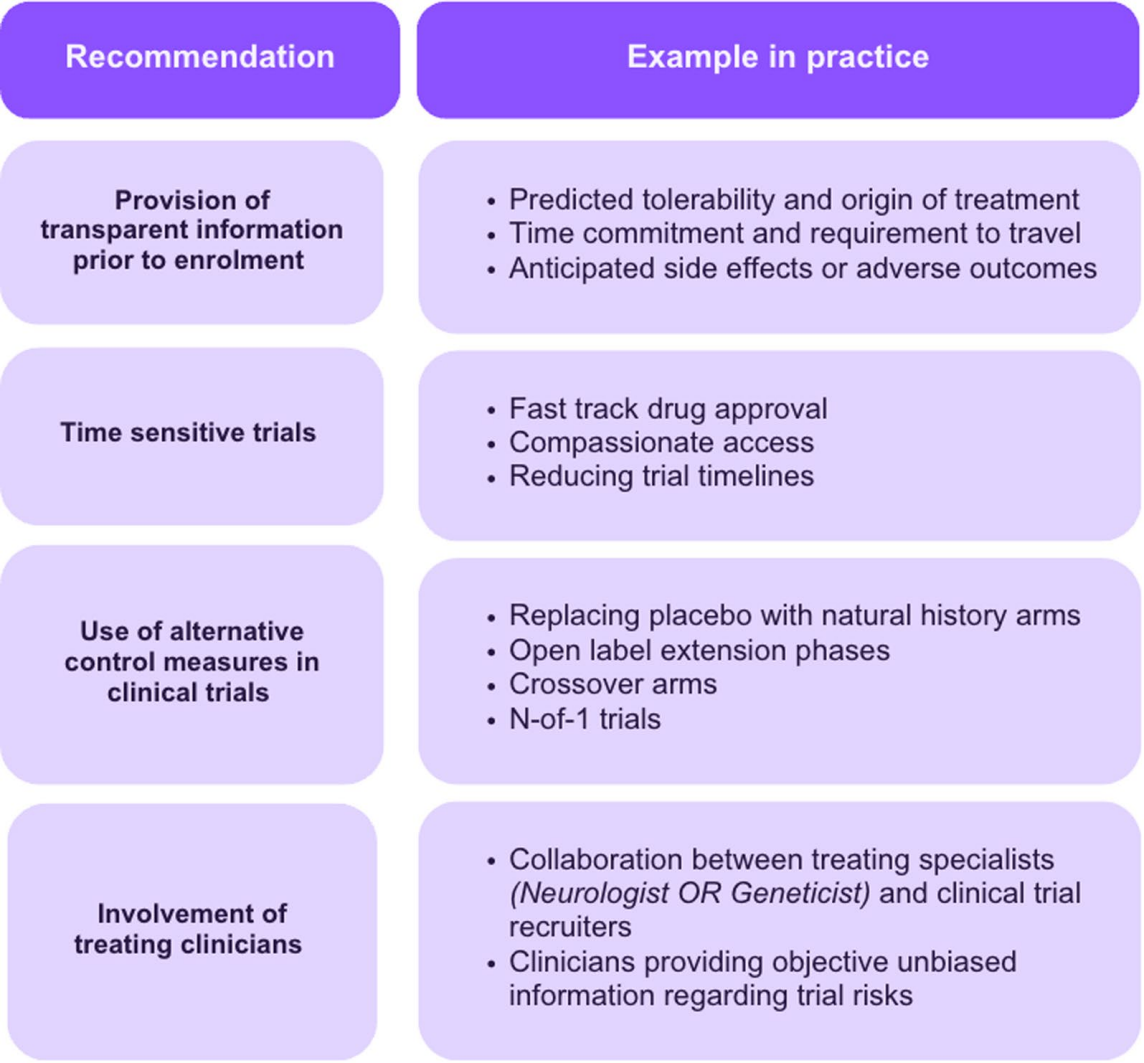
Facilitating recruitment into clinical trials—patient led recommendations

The below figure summarises patient led recommendations for the design of future Leukodystrophy clinical trials (Fig. 3).

### Limitations

These findings based on a small group of Australian patients should not be generalised to all individuals with Leukodystrophy.

Several participants recruited through the Australian Leukodystrophy Registry were known clinically to members of the research group. There is potential for this to have influenced what participants were willing to share in interviews. To protect the confidentiality of these participants, interviews were conducted by a separate member of the research team.

### Future directions

Having established patient led recommendations for future leukodystrophy trials, research efforts could be directed towards integration of said recommendations (Fig. 3) in the trial design process.

Further exploration of participants tendency for therapeutic optimism is also warranted to ensure accurate perception of clinical trial risks in leukodystrophy patients and their carers.

## Conclusion

This study adds to the body of literature surrounding clinical trial acceptability in rare disease cohorts. To capture the perspectives of individuals with leukodystrophy and their carers towards clinical trials, semi-structured explorative interviews were conducted.

Clinical trials appear to evoke hope, but access must be timely. The risk of adverse outcomes is a concern for prospective participants, however the perceived benefits of improved prognostic outcomes appeared to outweigh perceived harms. Further, there was a strong desire for individuals with leukodystrophy to access novel treatments. To support enrolment of leukodystrophy patients into future clinical trials, we suggest inclusion of treating clinicians in the recruitment process, provision of transparent information regarding the origin and anticipated side effects of treatments, compassionate use and fast-tracked drug approval, and the integration of alternative control measures in trial design.

Research efforts should now focus on translating patient led recommendations into trial design, as well as appropriate risk communication in trials to address therapeutic optimism and thus risk misinformation in leukodystrophy patient and their carers.

## Data Availability

The data that support the findings of this study are not openly available due to reasons of sensitivity and participant privacy however are available from the corresponding author upon reasonable request. Data is located within protected storage software (NVivo Version 1.6.2) with controlled access only by primary researcher EW.

## Abbreviations

CNS: Central Nervous System
VWM: Vanishing white matter disease
